# Basic Plastic Surgery Skills Training Program on Inanimate Bench Models during Medical Graduation

**DOI:** 10.1155/2012/651863

**Published:** 2012-12-30

**Authors:** Rafael Denadai, Andréia Padilha Toledo, Luis Ricardo Martinhão Souto

**Affiliations:** ^1^Institute of Plastic and Craniofacial Surgery, SOBRAPAR Hospital, Avenue Adolpho Lutz 100, Caixa Postal 6028, 13084-880 Campinas, SP, Brazil; ^2^Division of Plastic and Reconstructive Surgery, Department of Surgery, School of Medical Sciences, University of Marίlia (UNIMAR), 17525-902 Marίlia, SP, Brazil; ^3^School of Medical Sciences, University São Francisco (USF), 12916-900 Bragança Paulista, SP, Brazil

## Abstract

Due to ethical and medical-legal drawbacks, high costs, and difficulties of accessibility that are inherent to the practice of basic surgical skills on living patients, fresh human cadaver, and live animals, the search for alternative forms of training is needed. In this study, the teaching and learning process of basic surgical skills pertinent to plastic surgery during medical education on different inanimate bench models as a form of alternative and complementary training to the teaching programs already established is proposed.

## 1. Introduction

Recently there has been tremendous growth of ambulatory surgical procedures that general practitioners need to perform in order to treat cutaneous lesions [1–3]. In this context, as a large percentage of medical students do not acquire basic surgical skills during their training [4] and most of the general practitioners that perform ambulatory surgeries received no formal surgical training [5], it is necessary to establish a training program to teach and refine the basic surgical skills related to plastic surgery (e.g., to biopsy a cutaneous lesion and to reconstruct the defect by the rotation of a surgical flap) that are essential to perform these ambulatory surgical procedures during medical education [4–6].

Considering that surgical training on living patients (traditional learning) violates ethical and medical-legal aspects, that training on live animals and fresh human cadaver increases the risk of infections, involves high costs and limited access, requires specialized installations, and also contravenes ethical legal aspects, and that using virtual reality simulators involves high costs and restricted access [7, 8], the simulation-based basic surgical teaching on inanimate bench models is becoming widely used [9]. However, to date, it has not been established a teaching program that allows surgical skills to be completely acquired [4, 5], and new opportunities in simulation-based surgical education need to be explored to positively impact quality and safety in surgical care [10].

Among all the surgical specialties, plastic surgery now occupies a negligible component of many undergraduate curricula, and there is much discussion in the worldwide literature regarding if there is a place for plastic surgery in the undergraduate curriculum [11–14]. Moreover, plastic surgery as a specialty is poorly understood by medical students and healthcare professionals [11, 15–18], and one of the important reasons for this is limited and inadequate plastic surgery exposure at undergraduate level [15–17]. Although undergraduate exposure is an important influential factor for subsequent career interest in plastic surgery [16, 19], many medical students are in favor of having plastic surgery teaching even though many may not necessarily want to pursue a career in the specialty [20]. So, as teaching undergraduate plastic surgery has potential benefits to all future physicians and ultimately patients, irrespective of career intentions [17], some authors [13, 14, 21, 22] have reported the need for plastic surgery education at undergraduate level. 

Given the difficulties of changing the undergraduate curriculum [13, 23], simple solutions to increase plastic surgery exposure are required [13, 17]. Therefore, the aims of this study were to propose and to describe the teaching and learning process of basic surgical skills pertinent to plastic surgery during medical education using different inanimate bench models as a form of alternative and complementary training to the programs already established.

## 2. Simulation-Based Basic Plastic Surgical Skills Training

The proposal is based on self-directed training and feedback from instructors, distributed in several sessions (days, weeks, or months) of teaching and learning, interspersed with periods of rest [24, 25]. Each session consists of steps to be undertaken in subsequent ways: verbal teaching supervised by instructor and based on textbooks, online text, and online narrated expert demonstration videos; self-directed training on bench models with immediate feedback from the instructor in the classroom (or laboratory of simulation); self-directed training on bench models with posterior feedback from the instructor focused on extra-class procedures (the undergraduate must bring the bench model with the procedures carried out so that specific technical factors are assessed and constructive feedback is provided) [24–29].

## 3. Learning Goals

Once the basic skills training can lead to improved performance of more complex tasks [24], it is important to include teaching goals that are set before the beginning of the teaching and learning process in order of increasing difficulty, and these should be distributed in different training sessions [30]. Thus, as the student acquires simpler skills, more complex skills should be incorporated into the training. Initially, the goals may be similar for all group members. However, in subsequent sessions, proposals should vary according to individual needs. During the training steps, the instructor should explain the advantages and disadvantages of each technique, the proper choice of surgical materials, and proper use of surgical instruments. 

**Figure 1:**
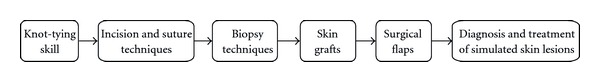
Learning goals. Undergraduates should initially perform basic surgical knowledge and then be trained on the most complex surgical skills.

In this training program, the basic plastic surgical skills are included (Figure 1) according to the analysis of the program for simulated training of surgical skills of the American College of Surgeons Program for Accreditation of Education Institutes performed by Rosen et al. [31].

## 4. Inanimate Bench Models

In recent years, different inanimate bench models have been proposed, discussed, and evaluated by our group [32–39] and by others [40–49]. In this training program, we adopted six inanimate bench models as teaching platforms (Table 1) because these enable the understanding of tridimensional procedures and also allow undergraduates to learn to respect the different layers of the skin (epidermis, dermis, subcutaneous cellular tissue, and muscles) during practice [32–49]. Such materials can be easily purchased from commercial outlets, such as craft shops and supermarkets. The parts of *postmortem* animals and organic materials must be gotten fresh and stored in refrigeration to reduce the risks of infections and increase the feasibility time of models.

## 5. Surgical Knots

The surgical knots (interlace made between the ends of a tread in order to unite and fix them) should be part of the simulated surgical teaching because they are essential for hemostasis and synthesis (key surgical times). The knots can be performed with the aid of instruments or manually (one or two hands), such as the nodes of the index finger (second finger), the middle finger (third finger), of surgeon, and of shoemaker. The manual knots must follow these principles: (a) equal movements of opposed hands perform a perfect knot; (b) the tip of the tread that changes its side after the first semi-knot should return to the initial side to perform another semi-knot; (c) the knots should be firm but without tension on the tissue (*in vivo*, excessive strain can result, for example, in avulsion of a blood vessel) [50, 51]. The different types of surgical knots should be practiced repeatedly until they can be performed quickly, effectively and almost automatically [52].

## 6. Incision and Suture Techniques 

**Figure 2:**
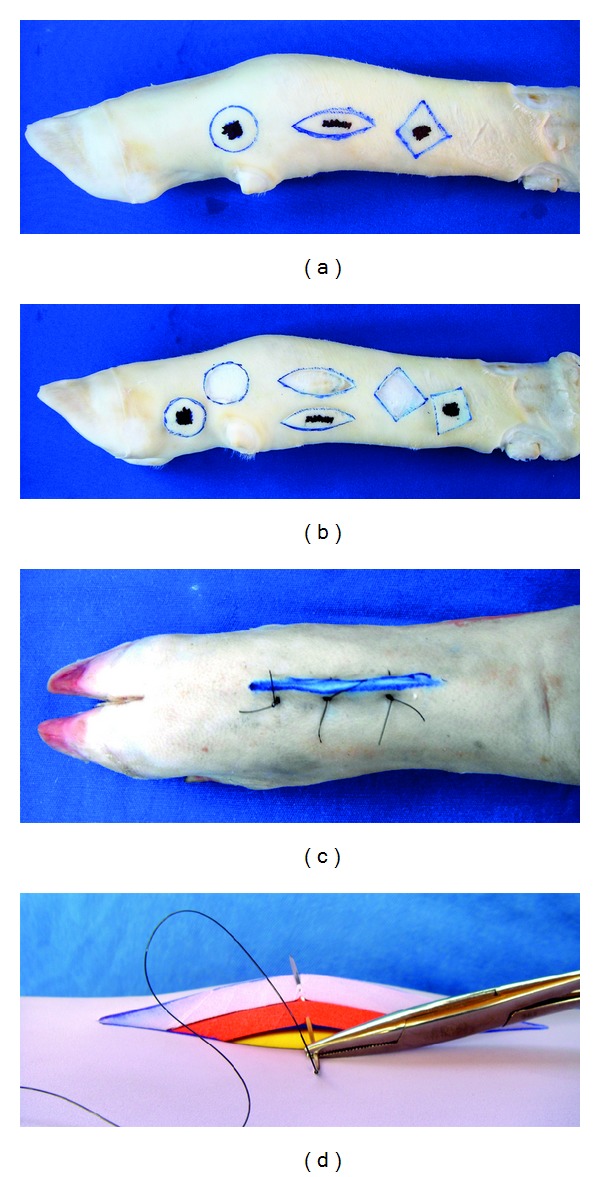
Inanimate bench models simulating incision and suture techniques. (a, b) Cattle-skin bench model simulating circular, linear and elliptic patterns of incision. (c) Pig-skin bench model simulating vertical mattress suture. (d) Synthetic ethylene-vinyl acetate bench model simulating subdermal interrupted suture; training should preferably be carried out near the edges of the material, and it is advisable to use multiple overlapping synthetic material plates aiming to mimic the different layers of the skin. Note that all the three bench models are simulating the procedures in a three-dimensional way.

The training of incisions (linear, circular, elliptical, vertical, and horizontal) and different sutures, such as simple interrupted sutures, vertical mattress suture according to Donati and McMillen, modified vertical mattress suture according to Allgöwer, horizontal mattress sutures, half-buried mattress sutures, subdermal interrupted sutures, running simple suture, running locked suture, and running subcuticular suture, can occur simultaneously (Figure 2). First, the undergraduate should mark the chosen material. The model is incised with the scalpel, which facilitates teaching the proper way to grip the instrument, its position with the “skin” (cutting angle between 30° and 60°), the way of the cut (firm and without “sawing” movements), and the depth of the incision [53, 54]. Following this, the created defects are repaired by placing points, also applying the technical aspects that are important to promote good healing, such as meticulous handling of tissues, proper positioning of the needle in the needle holder, angle of needle entry in the “skin,” exit of the needle in an equidistant point in relation to its entry, and approximation and eversion of the “wound edges” with proper tension [5, 55].

## 7. Biopsy Techniques 

**Figure 3:**
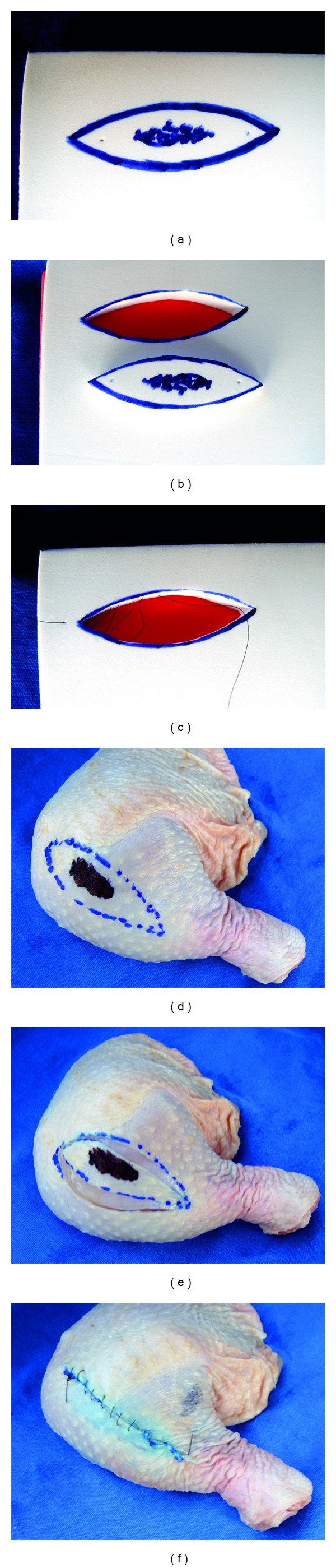
Inanimate bench models simulating elliptical biopsy technique. (a, b, and c) Synthetic ethylene-vinyl acetate bench model and (d, e, and f) chicken-skin bench model simulating (a, d) the safety margins forming an ellipse, (b, e) the intact removal of the “surgical piece", and repair of the surgical defects with the confection of (c) intradermal suture and (f) running simple suture. Note that both bench models allow three-dimensional understanding of the whole process of training.

The training of biopsy techniques (elliptical and circular; excisional and incisional; with and without safety margins) should be performed according to the previously set requirements. For example, for the practice of the classical elliptical incision (Figure 3), students should receive the following instructions [28, 53, 56–58].

**Drawing of the Ellipse**

The ellipse must be formed by two arcs that should be symmetrical in relation to the midline that separates them, and they should meet at the ends forming a convexity; the used curvature should be based on a length-width ratio of 3 : 1 to 4 : 1; a 30° angle should be used at the ends of the ellipse (intersection of the arcs).

**Safety Margins**

A line should be marked around the periphery of the “skin lesion” to delimit the safety margins; according to current recommendations for surgical resection of most cases of nonmelanoma skin cancer, the safety margins should be of 2 to 10 mm.

**Incision and Excision**

Smooth movements with the scalpel (cut angle between 30° and 60°), cuts of “subcutaneous tissue” with 1 or 2 movements, handling the tissue gently to avoid damaging the ellipse edges and the “epidermis,” and resection of the same amount of “tissue” in all areas of the “wound” should be done.

## 8. Skin Grafts 

Faced with a “skin” defect, students should plan a stamp graft in mesh or in strips with different diameters and thicknesses [59]. The graft should be removed intact from the donor area with a scalpel blade, Blair knife, or dermatome [46, 49, 59–63]; undergraduates should be trained on different pressures on the tissues and angulations between the blade and the “skin” in order to fabricate grafts of varying sizes and thicknesses [60–62]. After obtaining the graft, it should be placed and shaped in the receiving area so that the edges are well coadapted in all sides of the recipient area. Subsequently, the proper fixation of the graft should be carried out in order to reduce the dead space [59]. The simulation of the compressive dressing for skin grafts should also be part of the training [49].

## 9. Surgical Flaps

**Figure 5:**
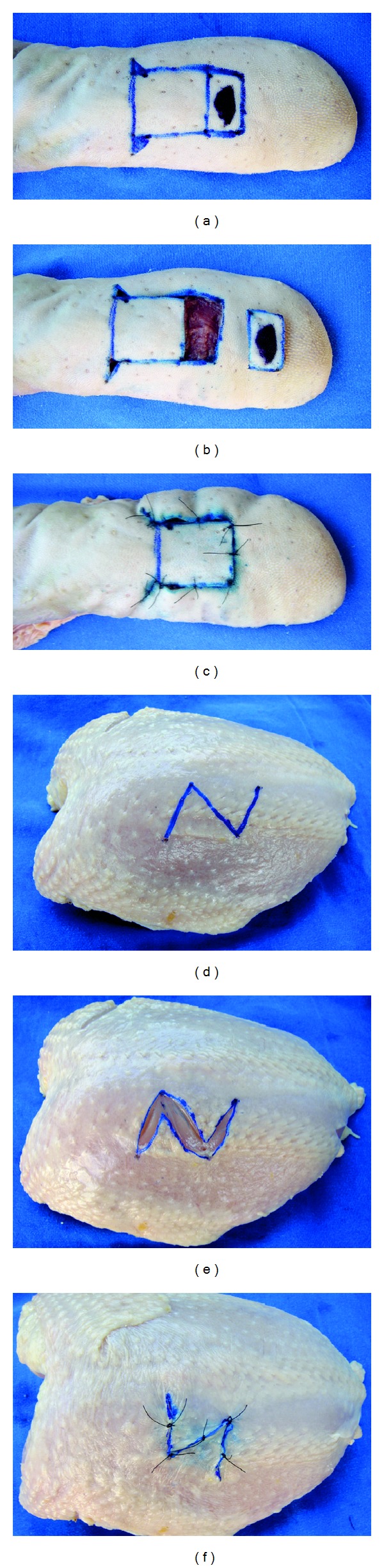
Inanimate bench models simulating flaps. (a, b, and c) Ox tongue bench model simulating a monopedicle advancement flap. (d, e, and f) Chicken-skin bench model simulating a Z-plasty. Note that students can easily see the advancement and transposition of surgical flaps, which often is hard to understand with the use of two-dimensional models. For example, it is simpler to explain that the center line of the Z-plasty should be placed along the scar, since it is this component that will be lengthened.

The bench models also allow the simulated practice of surgical flaps, such as transposition flaps (Z-plasty, W-plasty, rhomboid, and bilobed), of rotation, of advancement (V-Y and R-plasty), and in island (Figure 5). Faced with a “skin” defect, the carrying out of a flap based on schemas is planned [64]. From this, the markings are incised, the flap is moved to fill the defect, and simple stitches should fix the flap carefully, avoiding strain on its pedicle [65].

## 10. Diagnosis and Treatment of Simulated ****Cutaneous Lesions

Assuming the fact that the training of a complete procedure can be broken down into several components [66], following the acquisition of techniques of surgical knots, incisions, sutures, biopsies, grafts and flaps, the undergraduates can be trained on the diagnosis and treatment of simulated skin lesions by joining the learned skills. At this time, different “skin lesions” should be simulated on bench models, so that students make their respective diagnoses and/or treatments by using the previously learned principles and, then, the proper surgical repair. At this stage of the training, instructors should provide students with the cognitive aspects of decision making, such as which surgical procedure should be adopted in every kind of “skin lesion.” Different skin lesions can be simulated on bench models.

**Lipomas and Epidermoid Cysts**

**Figure 4:**
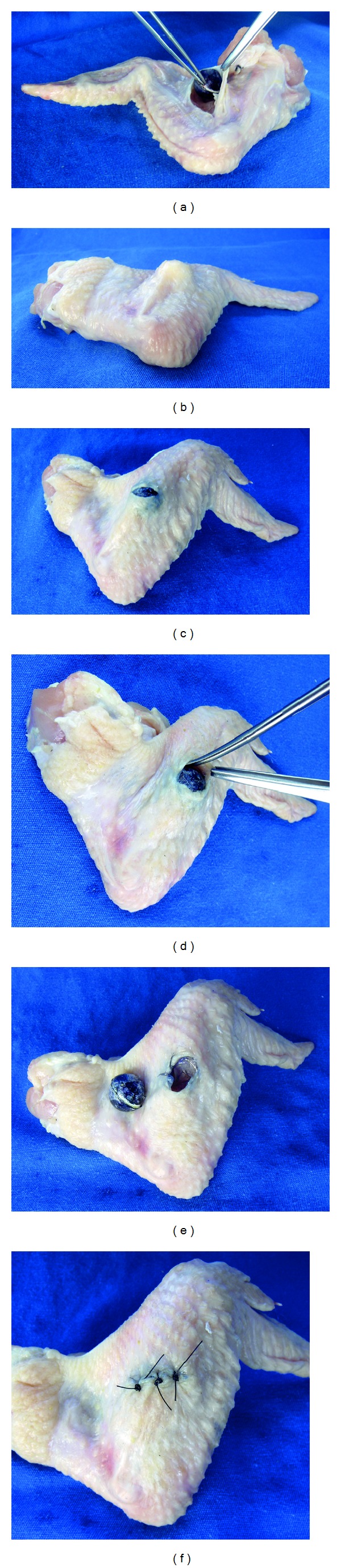
Chicken-skin bench model simulating a subcutaneous lipoma. (a) A small styrofoam ball should be placed in a subcutaneous tunnel made in the posterior portion of the model with the intention of (b) mimicking the cutaneous lesion. Following, students must (c) incise the skin, (d) carefully dissect the lesion, (e) resect it completely, and (f) repair the defect by means of single interrupted sutures.

To simulate these lesions, styrofoam balls, mini-balloons filled with ink or projectiles of paintball. should be inserted through a subcutaneous tunnel on parts of *postmortem* animals bench models [67, 68]. Undergraduates must respect the simulated lesion completely, taking care not to leave parts of the lesion in the wound (Figure 4).

**Necrotic Wounds**

**Figure 6:**
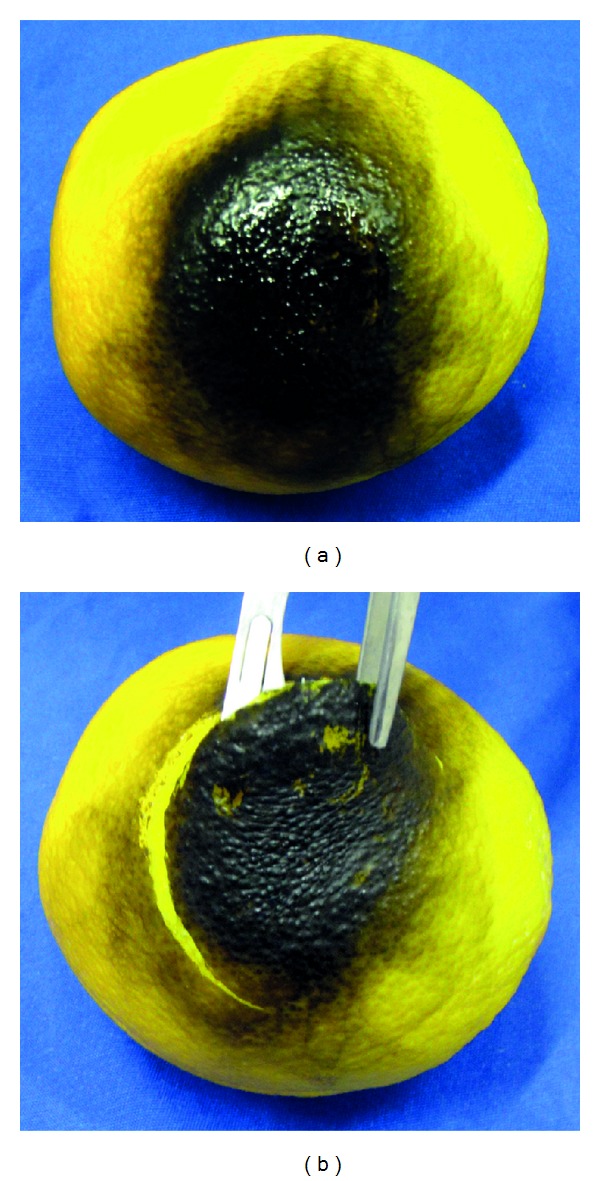
Organic bench model simulating (a) a necrotic wound and its (b) careful surgical debridement.

For training of tangential excision and surgical debridement (or escharotomy), the surface of the chosen bench model should be burned to simulate a necrotic area [69]. Undergraduates must respect the necrotic “tissue,” taking care not to damage the healthy tissue (Figure 6).

**Nonmelanoma Skin Cancer**

**Figure 7:**
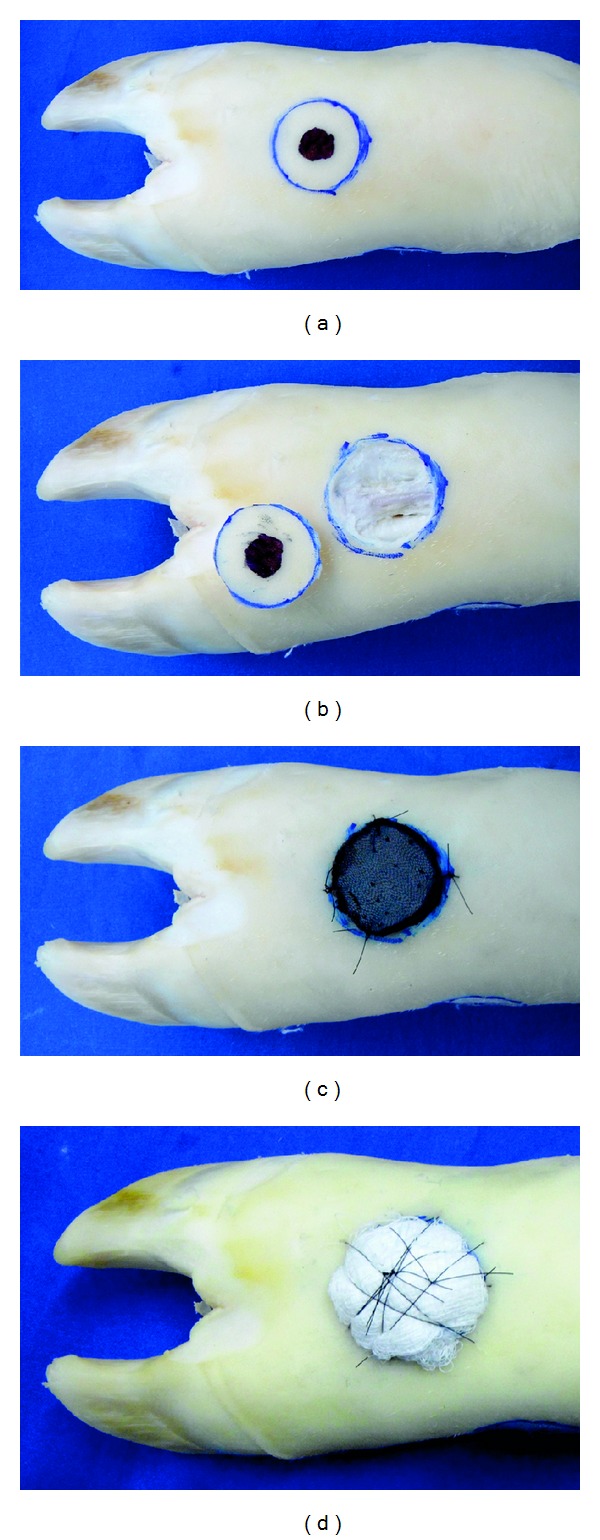
Cattle-skin bench model simulating (a) a nonmelanoma skin cancer with safety margins, (b) complete surgical excision of the “cutaneous tumor,” (c) repair of the defect by placing an ox tongue graft, and (d) a pressure dressing fixed with braided suture over the gauze wad.

The student should make an excisional biopsy with predetermined safety margins, since this is considered the standardized diagnostic therapeutic procedure for most cases of nonmelanoma skin cancer [58]. After the resection of different simulated skin lesions, undergraduates must make the appropriate repair of the created defect (primary approximation of the wound edges with stitch placing, graft or rotation, transposition or advancement of a flap) (Figure 7).

## 11. Training Time

There are no clear recommendations on the total number of hours that medical students must practice to acquire basic surgical skills. In this sense, in this basic plastic surgery training program, the number of training hours was distributed according to the complexities of skills (i.e., a longer training for those skills considered more complex) (Table 2). In general, the first week serves to introduce the subject (e.g., teaching issues such as clinical applicability of skills) and the other for the simulated training itself (hands-on training). It is important to take a break of one week between each of the six skills, totaling therefore six months of basic plastic surgery training (24 weeks, being 19 of teaching and learning and five of rest). A specific week or the total of weeks of a skill can be adjusted (i.e., increased or decreased) according to the individual or subgroups needs.

## 12. Self-Directed Training

During each training session, supervised by an instructor and at home, students should use the principles taught in an individualized, deliberate, repetitive, and participative way [30, 70]. Whenever there are doubts about a complete procedure or a particular step, they should seek for help from textbooks, online text, online narrated expert demonstration videos, and instructors [26–29].

## 13. Feedback

In the context of the acquisition of surgical skills based on simulation, feedback from instructors is associated with a better and faster learning and also with greater knowledge retention over time [30]. Thus, all undergraduates should receive feedback during and at the end of each training stage [30] in the classroom, or in specific times scheduled after the training at home [32–39]. Instructors must analyze specific movements, paying attention to inadequacies (e.g., mark lines and procedures already finished can serve as evaluation parameters), and following this, they should provide a constructive feedback (point and correct any technical errors) to students [71]. Thus, undergraduates improve skills based on their mistakes and can be trained again and again, having, as a result, the gain of skills over time. Concurrent with the feedback, it is important to encourage students to resolve their doubts during practice and after extracurricular tasks. 

To facilitate feedback, students should be distributed around rectangular tables, providing mobility to the instructor to clarify any doubts individually and also in subgroups [32, 42, 45].

## 14. Instructors

Feedback can be given by physician instructors (faculty expert or residents) and/or by nondoctors since they are qualified, such as laboratory technicians or medical students (monitoring format) [10, 24, 72, 73], without compromising the learning [72, 73]. The adoption of one instructor for each group of four undergraduates is recommended [74].

## 15. Assessment and Certification

Under simulated surgical teaching, we must emphasize the importance of an objective evaluation during and at the end of the whole teaching and learning process of each proposed surgical skill in order to measure the level of acquisition of the taught skills [75]. 

Among the various forms of the described assessments [31, 75], the Objective Structured Assessment of Technical Skills (OSATS) [76, 77] is currently considered as the gold standard for the objective evaluation of acquisition of surgical skills [75]. OSATS consists of two subscales: Task-Specific Checklist and Global Rating Scale (GRS) [76, 77].

Since GRS [76, 77] (Table 3) has the advantage of being used to assess generic aspects of technical performance and has a broad applicability, without the need to develop specific lists for each procedure [78], this scale has been adopted as a measurement and certification tool by our group [32–39] and by others [53, 78]. With this scale, it is possible to evaluate the performances of students in eight main areas, through a 5-point scale, being 1 the minimum score and 5 the maximum one, so that the maximum score achieved is 40 [53, 76, 77]. Instructors can apply this scale at the end of each training session, and in subsequent sessions, they follow the gain of skills and specific points (among the eight evaluated ones) that deserve attention.

In addition, GRS [76, 77] can also be used as a certification tool; for an individual task, the candidate should achieve a score of 24 or more to be considered competent [78]. Therefore, if the trainee meets the predefined criteria based on objective assessment, he/she can progress to the next stage of training (considered as a more complex one). However, if the undergraduate is not able to proceed, the training should be repeated and focused on specific deficits, and, then, a new objective assessment should be carried out.

In the training evaluation, a characteristic of bench models that could be considered as a problem (they can tear) is actually an advantage because this occurs when students make a wrong movement (e.g., applying excessive force). This characteristic can serve as an evaluation mechanism with feedback for improving skills [32–34]. Moreover, the markings made on the surfaces of models also serve as an evaluation parameter [33].

## 16. Discussion

Over the last two decades, simulation-based education has emerged as an important innovation in medical learning and practice [31, 79]. In this context, surgical training is shifting from the traditional apprenticeship to a more objective standardized approach, using simulators to improve several medical aspects such as reducing errors and increasing patient safety [31]. Since currently the simulated acquisition of basic surgical skills is recommended before any procedures on living patients [7], the main focus of this study was to propose a simulation-based basic plastic surgery training program during medical education, through the training on different inanimate bench models. In order to increase the arsenal of surgical skills of medical students during training, this teaching proposal and the way bench models are applied can be incorporated and adapted to complement the curriculum already established in different educational institutions [8, 30], and this,can be used in several disciplines such as surgical technique, plastic surgery, among others. Both novice medical students and students that master basic surgical skills partially, but that need to improve them, can benefit from this program. Similar to other studies [13], this training program also has the potential to introduce and improve students' plastic surgery skills, as well as develop personalcareerinterests.

Based on the assumption recently described as the most effective method to teach surgical skills in simulation environments, a combination of self-directed training with instructors' feedback, intermittently distributed over a predetermined period (weeks or months) [24, 25], this was the teaching form adopted in the present study in order to retain and improve the learned surgical skills [24, 25]. However, some factors, such as high costs [25] (mainly in developing countries) [32, 33, 43], the lack of time, and shortage of faculty experts (traditional instructor) [10, 24] have been described as limiting factors for the implementation of this simulated training strategy. 

One solution to partially reduce the financial cost is the use of low cost bench models, such as those described in the present study. The different inanimate bench models vary in relation to the fidelity level (realism) when compared to a live human being; there are high fidelity models, such as parts of *postmortem* animals (pig [37, 38, 40, 45, 46], chicken [38, 39, 44], and cattle [48] skins and ox tongue [40–42]) and others of low fidelity, such as plates of ethylene-vinyl acetate [32, 34–38, 47], organic material [33, 49], among others [40, 53].

Despite the intuitive belief that “the more realistic, the best,” in the simulated training of surgical skills, the acquisition of skills should be measured by means of an objective method [75]. Therefore, since there are studies developed by our group [36–38] and by others [80–83] that demonstrate objectively that the surgical skills learned by novice undergraduate on bench models can result in improved performance in animals, corpses, and also in the operating room, regardless of the fidelity of bench model [9, 38, 39, 80–83], the choice of a specific bench model should not be based on its fidelity. Aspects such as availability, seasonal variability, and costs should be considered for this choice.

 The authors believe that the bench models are complementary. In order to generate the interest of medical students in the practice of plastic surgery principles, the initial training in classrooms (or laboratories of surgical technique) should be preferably performed on bench models made from parts of *postmortem* animals, because it was shown that students feel more attracted by these bench models [40]. For the subsequent training sessions, low-fidelity bench models should be preferred because they are versatile, reusable, and easy to handle [32, 33, 35, 47, 49], unlike the *ex vivo* model that requires adequate space and conditions to be stored [7, 8], and it can make the training impracticable, for example, at home [35].

Although financial costs can be reduced by the previously described measures, time availability remains a problem for faculty experts [10, 24]. Feedback generated by computers could be an option to reduce the supervised learning time. However, besides the high cost for its acquisition, the retention of skills over time is significantly greater when learned from direct feedback from an instructor [84]. Similar to that described here, the incorporation of residents, trained medical students, or nonphysician skills laboratory [10, 72, 73] as instructors is an alternative that can reduce the number of faculty surgeons transferred from patient care to simulation environments. With this measure, faculty surgeon would focus on teaching complex tasks and cognitive aspects of clinical training (e.g., decision making) that are not duties of the nonmedical instructor [73]. Alternatives that can also help reduce the time of supervised simulated teaching would be increased intervals between training sessions [85], to use concepts derived from blended learning [86], and to encourage the practice outside the classroom, for instance, at home, as it has been proposed by our group [32–39]. 

The present training program was structured especially to develop some basic plastic surgical skills. Therefore, it does not meet all the needs of medical students in training, which should include the acquisition of other basic surgical skills, as it is described by the American College of Surgeons/Association of Program Directors in Surgery National Skills Curriculum [31].

## 17. Conclusion

The proposal of simulation in basic plastic surgery training on inanimate bench models is a further complementary alternative to the arsenal of training programs already established in order to better prepare medical students before their contact with living patients which remains as the cornerstone of medical education.

## Figures and Tables

**Table 1:** Advantages and disadvantages of inanimate bench models [8, 9, 31–49] adopted as learning tools in this basic plastic surgery training program.

Inanimate bench models	Fidelity	Infection risk	Financial costs*	Availability*	Easy handling	Reutilization**
Parts of *postmortem* animals						
Ox tongue	High	Present	+++	Variable	++	Possible
Cattle skin	High	Present	+++	Variable	++	Possible
Pig skin	High	Present	+++	Variable	++	Possible
Chicken skin	High	Present	+++	Variable	++	Possible
Organic material						
Fruits and vegetables	Low	Present	++	Variable	+++	Possible
Synthetic material						
Ethylene-vinyl acetate	Low	Absent	+	Variable	+++	Unlimited

*Varies according to seasonality and geographical region; **limited by risk of infections and natural deterioration of material.

**Table 2:** Proposed training time in this basic plastic surgery training program.

	Training time (h) per week				
Learning goals	First day		Second to sixth day	Seventh day	Total number of weeks**
	Verbal teaching based on theoretical materials	Training on BM in the classroom (process feedback)	Training on BM outside the classroom*	Training on BM after practice outside the classroom (outcome feedback)
Surgical knots	1	1	Variable	1	2
Incision and suture techniques	1	2	Variable	2	2
Biopsy techniques	1	3	Variable	3	3^*≈*^
Skin grafts	1	3	Variable	3	3^¥^
Surgical flaps	1	4	Variable	4	5^o^
Management of cutaneous lesions	2	4	Variable	4	4^#^

h: hour; BM: bench models; *each medical student must train repeatedly for as long as you feel necessary; instructor's role is to encourage this practice outside the classroom; **one week for introduction of the subject in each of the six skills; ^*≈*^one week for incisional biopsies (without safety margins) and one for excisional biopsies (safety margins); ^¥^one week for the handling of the surgical instruments for the preparation of graft (donor area) and one for graft placement on recipient area; ^o^one week for each type of flap (transposition, rotation, advancement, and island flaps); ^#^one week for each proposed cutaneous lesion (lipomas/epidermoid cysts, necrotic wounds, and non-melanoma skin cancer).

**Table 3:** Global rating scale adapted to evaluate the sutures and biopsies techniques [53, 76, 77].

Please rate the trainee's performance on the following scale.					
Respect for tissue	1	2	3	4	5

	Frequently used unnecessary force on tissues or caused damage by inappropriate instrument use		Careful handling of tissue, but occasional inadvertent damage		Consistently handled tissues appropriately with minimal damage

Time in motion	1	2	3	4	5

	Many unnecessary moves		Efficient time and motion, but some unnecessary moves		Clear economy of movement and maximum efficiency

Instrument handling	1	2	3	4	5

	Repeatedly makes tentative or awkward moves with instruments		Competent use of instruments, but occasionally awkward		Fluid movements

Elliptical excision skill*	1	2	3	4	5

	Lacks knowledge of design parameters (<2 mmor >10 mm margins); angles *very *different than 30°; length-width ratio *very *different than 3-4 : 1		Adequate 2 to 10 mm margins; angles at ends of ellipse *slightly *different than 30°; length-width ratio *slightly *different than 3-4 : 1		Adequate 2 to 10 mm margins; 30° angles at both ends; length-width ratio 3-4 : 1

Suture training**	1	2	3	4	5

	Awkward and unsure with poor knot tying, and inability to maintain tension		Competent suturing with good knot placement and appropriate tension		Excellent suture control with correct suture placement and tension

Flow of operation	1	2	3	4	5

	Frequently stopped operating, seemed unsure of next move		Demonstrated some forward planning and reasonable progression of procedure		Obviously planned operation

Knowledge of procedure	1	2	3	4	5

	Inefficient knowledge of procedure. Looked unsure and hesitant		Knew all important steps of procedure		Demonstrated familiarity of all steps of procedure

Final product	1	2	3	4	5

	Final product of unacceptable quality		Final product of average quality		Final product of superior quality

Overall performance	1	2	3	4	5

	Very poor		Competent		Very good

Maximum total score (40)					

Total score ( )					

*This parameter should be excluded for the evaluation of suture techniques; **this parameter should be excluded for the evaluation of biopsy techniques.
